# La Non-Alcoholic Fatty Liver Disease, una patologia epatica di interesse endocrinologico

**DOI:** 10.1007/s40619-021-00955-9

**Published:** 2021-09-15

**Authors:** Elena Gangitano, Stefano Ginanni Corradini, Carla Lubrano, Lucio Gnessi

**Affiliations:** 1 Dipartimento di Medicina Sperimentale, Sapienza Università di Roma, Policlinico Umberto I, Roma, Italia; 2 Dipartimento di Medicina Clinica, Sapienza Università di Roma, Policlinico Umberto I, Roma, Italia

**Keywords:** NAFLD, Steatosi epatica, Ormoni, Metabolismo

## Abstract

La Non-Alcoholic Fatty Liver Disease (NAFLD) comprende diversi stadi di patologia epatica che vanno dalla steatosi alla cirrosi epatica. È considerata la manifestazione epatica della patologia metabolica e alcune alterazioni endocrinologiche possono concorrere a determinarne l’insorgenza o la progressione. È quindi di fondamentale importanza che l’endocrinologo sia consapevole dell’importanza clinica e prognostica della NAFLD correlata alle patologie endocrine, al fine di offrire al paziente il trattamento adeguato.

**Informazioni Supplementari:**

La versione online contiene materiale supplementare disponibile su 10.1007/s40619-021-00955-9.

## Introduzione

**Figure 1:**
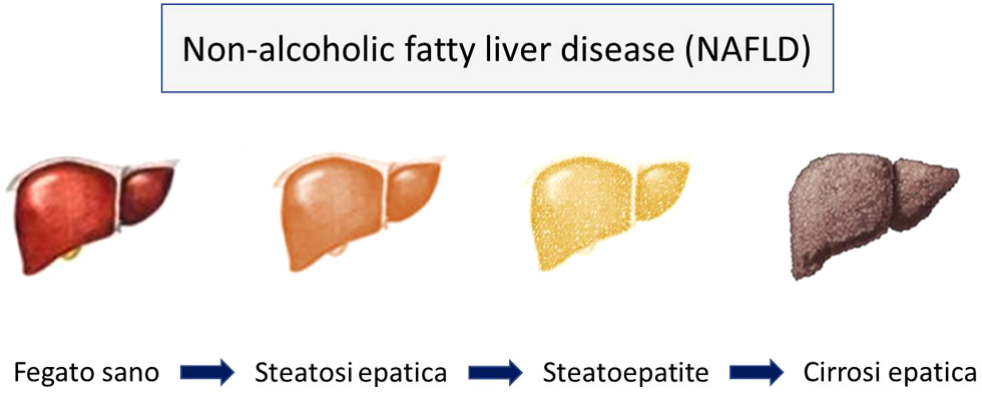

La Non-Alcoholic Fatty Liver Disease (NAFLD) è considerata la manifestazione epatica della patologia metabolica, e consiste in uno spettro di patologie epatiche che comprendono l’accumulo epatico di lipidi in più del 5% degli epatociti (steatosi), l’infiammazione epatica (steatoepatite), la fibrosi, la cirrosi e lo sviluppo di epatocarcinoma (Figura 1), in assenza di consumo alcolico maggiore o uguale a 20 g nelle donne e 30 g negli uomini, e di altre cause secondarie [1].

La NAFLD costituisce attualmente un problema di salute pubblica: nella popolazione generale la sua prevalenza è stata stimata attorno al 25% ma le percentuali variano in relazione alle diverse popolazioni oggetto di studio, a causa dell’eterogeneità in termini di etnia, età e sesso, nonché dei metodi utilizzati per diagnosticarla. È stato stimato che fino al 75% dei soggetti diabetici possa presentare NAFLD, e tale prevalenza aumenta ulteriormente nei pazienti obesi [2].

La NAFLD si associa a una maggiore incidenza di patologie cardiovascolari, con cui condivide molti fattori di rischio metabolici. È stato infatti osservato che i pazienti con NAFLD presentano disfunzione endoteliale, aumento della rigidità delle pareti delle arterie e aumento dello spessore dell’intima-media carotidea, parametri riconosciuti come marker di patologia cardiovascolare [1].

Inoltre, la fibrosi avanzata nella steatoepatite (Non-Alcoholic Steato Hepatitis, NASH) è associata ad aumentata mortalità da cause cardiovascolari e, come dimostrato recentemente, da COVID-19 [3].

Per la NAFLD non esistono attualmente terapie specifiche. È noto dalla letteratura che il cambiamento dello stile di vita, focalizzato su calo ponderale, moderazione nell’assunzione di alcolici ed esercizio fisico, determinino un’importante riduzione dell’entità della steatosi epatica e dell’insulino-resistenza [1]. Tali misure di cambiamento dello stile di vita sono peraltro condivise per la prevenzione e il trattamento di altre patologie metaboliche, come il diabete mellito.

Un problema particolarmente rilevante è rappresentato dalla progressione della NAFLD in NASH e quindi in cirrosi, nonché il rischio di insorgenza di epatocarcinoma, che può verificarsi frequentemente anche negli stadi pre-cirrotici. I fattori determinanti la progressione da steatosi semplice a NASH non sono stati ancora completamente chiariti; di conseguenza, allo stato attuale non è possibile predire con certezza l’evoluzione della patologia. È stato riportato che lo stress ossidativo possa giocare un ruolo fondamentale nel determinare il danno epatocitario e quindi possa favorire tale progressione [4].

Secondo stime recenti e in considerazione dell’efficacia dei nuovi farmaci antivirali, la NAFLD rappresenterà la prima causa di trapianto di fegato in Europa nei prossimi anni: il suo impatto in termini di morbilità e mortalità non è quindi trascurabile.

## Fisiopatologia della NAFLD

I meccanismi alla base della genesi della steatosi epatica non sono ancora completamente chiariti. È noto che l’accumulo epatico di lipidi si associa a obesità viscerale, insulino-resistenza e dislipidemia, nel complesso quadro della sindrome metabolica. In alcuni studi è stato riscontrato che la steatosi epatica precedeva la manifestazione del diabete mellito e/o della sindrome metabolica, ipotizzando che la NAFLD possa quindi essere considerata un fattore di rischio per lo sviluppo di entrambe le condizioni [5].

La NAFLD è caratterizzata da un accumulo di trigliceridi all’interno degli epatociti sotto forma di gocce lipidiche intracellulari. I trigliceridi vengono costituiti dall’esterificazione degli acidi grassi liberi (FFA) e del glicerolo. L’aumentata disponibilità epatica di acidi grassi liberi è dovuta sia all’aumentato afflusso di acidi grassi al fegato per eccessivo apporto dietetico e alterata inibizione della lipolisi a livello del tessuto adiposo causata dall’insulino-resistenza, sia all’aumento della lipogenesi epatica *de novo*. Gli acidi grassi liberi possono andare incontro a $\beta $-ossidazione o esterificazione a trigliceridi, con immagazzinamento sotto forma di gocciole lipidiche o incorporazione nelle lipoproteine a densità molto bassa (VLDL). Uno sbilanciamento quindi tra sintesi, afflusso, ossidazione ed escrezione determina l’accumulo dei trigliceridi tipico della steatosi epatica [6]. L’accumulo di alti livelli di acidi grassi liberi e colesterolo libero, nonché di altri metaboliti lipidici, comporta lipotossicità, con conseguente disfunzione mitocondriale e stress ossidativo.

**Figure 2:**
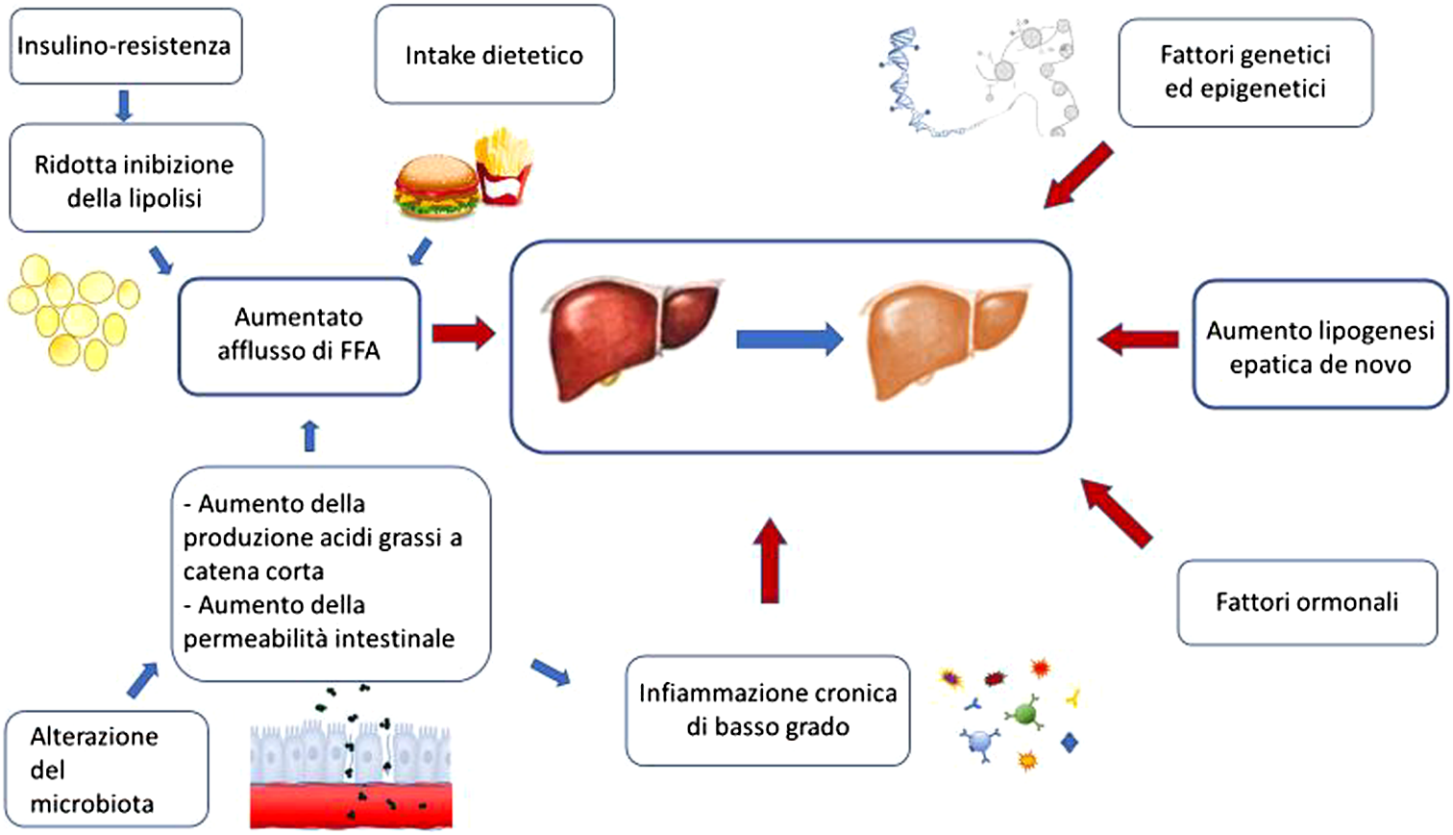

Secondo la teoria della patogenesi *multiple hit*, numerosi fattori interagiscono per determinare l’insorgenza di NAFLD in soggetti geneticamente predisposti (Figura 2); tali fattori includono dieta, insulino-resistenza, alterazioni del microbiota intestinale, predisposizione genetica e modificazioni epigenetiche [7, 8]. Per quanto riguarda il microbiota, l’alterazione della composizione e della capacità metabolica della flora batterica intestinale ha un effetto diretto sul fegato e uno indiretto, favorendo l’obesità e il diabete di tipo 2 [9]. Diversi metaboliti batterici, quali lipopolisaccaridi (LPS), acidi grassi a catena corta, colina, trimetilammina-N-ossido (TMAO), con meccanismi molto complessi regolano la permeabilità intestinale, l’appetito, l’accumulo di grasso e l’infiammazione [7, 9, 10].

Recentemente, proprio per la patogenesi così complessa della malattia, per il fatto che nel singolo paziente possa prevalere uno dei fattori patogenetici e che la gestione dei pazienti migliori grazie a una loro stratificazione in tal senso, è stata proposta una nuova terminologia per la NAFLD, ossia *Metabolic* (*dysfunction*) *associated fatty liver disease* (MAFLD) [8]. In altri termini, la nuova nomenclatura di MAFLD e, soprattutto, la stratificazione dei pazienti in sub-fenotipi secondo il fattore patogenetico prevalente permetterà di disegnare trial clinici terapeutici migliori per identificare nuovi farmaci.

Anche alcune alterazioni ormonali possono concorrere a determinare l’insorgenza della steatosi epatica, sia favorendo lo sviluppo della sindrome metabolica, sia determinando quadri di NAFLD secondaria [11, 12].

## NAFLD e patologie endocrinologiche

L’eccesso di glucocorticoidi è associato a un fenotipo metabolicamente compromesso, caratterizzato da obesità centrale, insulino-resistenza e diabete. Nella sindrome di Cushing è stata riscontrata una prevalenza di steatosi epatica non trascurabile, di circa il 20%, significativamente correlata al tessuto adiposo viscerale [13]. Uno studio ha osservato che i pazienti affetti da NAFLD presentavano una lieve iperattività dell’asse ipotalamo-ipofisi-surrene, e il cortisolo libero urinario delle 24 ore e il cortisolo dopo soppressione con 1 mg di desametasone risultavano essere predittori indipendenti di NAFLD e della severità della fibrosi epatica [14].

Si ritiene che i glucocorticoidi in eccesso, siano essi esogeni o endogeni, concorrano all’insorgenza della steatosi epatica mediante inibizione della $\beta $-ossidazione degli acidi grassi e promozione della lipogenesi epatica *de novo* [6].

Il GH influenza fisiologicamente il metabolismo glico-lipidico, promuovendo la lipolisi, la glicolisi e la gluconeogenesi, ma è altresì responsabile di un aumento dell’insulino-resistenza. Dati recenti suggeriscono una forte associazione tra i livelli epatici di IGF-1 e alcune proteine leganti l’IGF e il grado di steatosi epatica [15]. L’associazione tra il deficit di secrezione dell’ormone della crescita nell’adulto (GHD) e la steatosi epatica non è ancora completamente chiarita. Alcuni studi nei pazienti con GHD riportano alterazioni del profilo glico-lipidico e della composizione corporea, aumento dell’obesità viscerale e maggiore incidenza di sindrome metabolica mentre, al contrario, altri studi non hanno rilevato alcuna differenza statisticamente significativa tra soggetti sani e pazienti affetti. Allo stesso modo, non tutti gli studi sono concordi nel riportare un aumentato contenuto di grasso a livello epatico nei GHD e non abbiamo dati univoci che riportino una chiara efficacia della terapia ormonale sostitutiva nel determinarne la riduzione [16, 17].

Recentemente, alcuni studi sull’animale hanno mostrato come la prolattina, tramite il legame al suo recettore PRLR, sia in grado di ridurre l’accumulo epatico di trigliceridi [18], fornendo le basi per la ideazione di un nuovo modello matematico, che include i valori di prolattina, per stimare la presenza e la severità della NAFLD nell’uomo [19].

L’associazione tra NAFLD e androgeni mostra un dimorfismo sessuale: la steatosi epatica si associa, infatti, a iperandrogenismo nelle donne e ipogonadismo negli uomini [20].

Nell’uomo, la prevalenza della NAFLD risulta aumentata nei soggetti con bassi livelli di testosterone circolante rispetto a soggetti con più alti livelli di testosterone circolante [12, 20]. L’efficacia della terapia sostitutiva dell’ipogonadismo nel determinare miglioramento dei parametri metabolici e riduzione del contenuto epatico di grasso, però, non è dimostrata in maniera univoca [21].

Le donne affette da sindrome dell’ovaio policistico (PCOS) presentano un’alta prevalenza di steatosi epatica, e ciò è particolarmente evidente nella PCOS con iperandrogenismo, che si associa a un fenotipo metabolicamente più compromesso [22]. Alcuni studi in donne in età post-menopausale hanno dimostrato un’associazione tra aumento dei livelli circolanti di testosterone libero e steatosi epatica [23].

L’ipogonadismo femminile gioca un ruolo altrettanto importante; gli estrogeni sembrano, infatti, svolgere un ruolo protettivo nei confronti della steatosi e della sua progressione [24].

Non è stato ancora completamente chiarito se l’ipotiroidismo giochi un ruolo nella genesi della NAFLD. Gli ormoni tiroidei hanno la capacità di influenzare il metabolismo lipidico; di conseguenza, la loro azione a livello epatico potrebbe potenzialmente influenzare la genesi della steatosi. Alcuni studi hanno messo in luce come l’ipotiroidismo, anche se subclinico, si associ a una maggiore prevalenza di steatosi epatica, anche dopo aggiustamento per età, BMI, diabete mellito e profilo lipidico. Altri studi in letteratura, invece, non hanno riscontrato tale associazione; di conseguenza, tale correlazione rimane ancora da definire [12]. D’altro canto, dati pubblicati di recente sostengono l’ipotesi che la NAFLD indotta dall’ipotiroidismo possa essere considerata un’entità clinica separata, suggerendo anche possibili opzioni di trattamento per la NAFLD attraverso la terapia sostitutiva per l’ipotiroidismo insieme a modifiche dello stile di vita. Inoltre, un intero nuovo campo di ricerca è focalizzato sui tiromimetici nel trattamento NAFLD/NASH, attualmente in fase 3 di sperimentazione clinica [25].

L’associazione di NAFLD e diabete mellito di tipo 2 è ben riconosciuta in letteratura e la prevalenza di NASH nei soggetti diabetici supera il 35% (Tabella 1). Il diabete, infatti, oltre a rappresentare un fattore di rischio per lo sviluppo di NAFLD, sembra accelerarne la progressione [26, 27]. La NAFLD, d’altro canto, costituisce un fattore di rischio sia per lo sviluppo di diabete mellito di tipo 2 che delle sue complicanze [27].

**Table 1:** 

Prevalenza della NAFLD nei pazienti affetti da diabete mellito di tipo 2 nel mondo	
Europa	68% (62,1–73)
Stati Uniti d’America	51,8% (31,3–71,6)
America Latina	56,8% (34,1–77)
Africa	30,4% (11,6–67,1)
Asia Occidentale	67,3% (60,4–73,6)
Asia Meridionale	57,9% (52,9–62,7)
Asia Orientale	52% (45,4–58,6)

Per quanto riguarda la prevalenza della NAFLD nel diabete di tipo 1, i dati presenti in letteratura non sono del tutto concordanti. Alcuni studi la riportano come relativamente bassa (<10%), probabilmente grazie alla soppressione della lipolisi ad opera della terapia insulinica, con conseguente riduzione dell’afflusso di acidi grassi liberi al fegato [2], ma una recente metanalisi riporta una prevalenza complessiva di circa il 20% [28]. Nei pazienti con diabete mellito di tipo 1 la steatosi determina un aumento del rischio cardiovascolare, similmente a quanto riscontrato nei soggetti sani e nei pazienti affetti da diabete mellito di tipo 2 [29].

Le alterazioni ormonali appena descritte, in particolare diabete mellito di tipo 2, GHD, ipotiroidismo e ipogonadismo, sono presenti in frequenza non trascurabile nei pazienti affetti da obesità, e possono contribuire all’insorgenza e al peggioramento della steatosi epatica in questi pazienti già predisposti allo sviluppo della NAFLD. L’endocrinologo rappresenta spesso il primo specialista a cui il paziente con patologia metabolica si rivolge, e la ricerca della NAFLD andrebbe considerata nell’ambito dell’iter di inquadramento diagnostico del paziente obeso complicato.

D’altro canto, l’obesità di per sé si può associare a un fenotipo metabolicamente compromesso o a un fenotipo metabolicamente sano, e il 30% degli individui normopeso può essere considerato metabolicamente obeso, in quanto presenta alterazioni metaboliche tipiche dell’obesità in presenza di un BMI non patologico. Ciò concorda con il fatto che una percentuale non trascurabile dei pazienti affetti da steatosi epatica non presenta eccesso ponderale, e tali soggetti magri con steatosi (*lean NAFLD*) presentano tratti metabolici e di flora batterica intestinale peculiari [8].

## Conclusioni

La NAFLD e alcune patologie endocrinologiche, come brevemente discusso, sono fortemente interconnesse. Come alcune alterazioni ormonali si associano a un’aumentata incidenza di steatosi epatica, al contrario il riscontro di NAFLD, soprattutto se severa, può suggerire la presenza di alterazioni endocrinologiche.

In considerazione degli effetti a lungo termine della NAFLD in termini di morbidità e mortalità, è fondamentale che l’endocrinologo sia consapevole dell’importanza di diagnosticarla e avvii un iter appropriato di monitoraggio a lungo termine e un approccio terapeutico integrato in accordo con i colleghi epatologi.

## Informazioni Supplementari

I link al materiale elettronico supplementare sono elencati qui sotto.

## Consenso informato

Lo studio presentato in questo articolo non ha richiesto sperimentazione umana.

## Studi sugli animali

Gli autori di questo articolo non hanno eseguito studi sugli animali.
