# Supplementary material for: La Non-Alcoholic Fatty Liver Disease, una patologia epatica di interesse endocrinologico
Source: L'Endocrinologo. 2021 Sep 15;22(5):436–40. [Article in Italian] doi: 10.1007/s40619-021-00955-9 (PMC8442522; doi:10.1007/s40619-021-00955-9)
Supplement: Supplementary file 2 [file 40619_2021_955_MOESM2_ESM.doc]

**RISPOSTE**

**1. Quali stadi di patologia epatica comprende l’acronimo NAFLD?**

b. steatosi epatica, steatoepatite, cirrosi epatica

**2. A quali patologie endocrinologiche è chiaramente associata la NAFLD?**

c. diabete mellito di tipo 2, ipogonadismo, insulino-resistenza

**3. Quali di questi fattori sono maggiormente implicati nella genesi della NAFLD?**

b. insulino-resistenza e sindrome metabolica

**4. Indicare la risposta errata. La NAFLD:**

b. è meno prevalente negli uomini affetti da ipogonadismo
